# Feasibility Study and Experimental Evaluation of the Design of Nodule Prototype Developed for Palpation Display Apparatus: A Novel Device for Contactless Primary Tactile Diagnosis

**DOI:** 10.3390/mi12050576

**Published:** 2021-05-19

**Authors:** Sakura Sikander, Pradipta Biswas, Sang-Eun Song

**Affiliations:** Department of Mechanical and Aerospace Engineering, University of Central Florida, Orlando, FL 32816, USA; pvbiswas@knights.ucf.edu

**Keywords:** palpation, soft medical device, tactile, soft robotics, stiffness, tumor, remote diagnosis, granular jamming, palpation simulator

## Abstract

Background: Lack of feasible palpation display for primary diagnosis of a tumor without any need of physician to patient physical contact has been reported as one of the major concerns. To further explore this area, we developed a novel palpation device consisting of a uniquely designed nodule mechanism (based on optimizing nodule top and bottom hemisphere wall thickness and manipulating granular jamming method) that can vary stiffness while maintaining the shape of the same nodule display, for which current devices are not capable of in terms of aping a tumor. Methods: This paper evaluates the manufacturing approach of the nodule, exploring several iterations of the nodule prototype. Experiments were performed on nodule prototypes of varying wall thicknesses in order to evaluate its effect on stiffness and deformation. Results and Conclusions: Experimental results showed that nodule top and bottom wall thickness had a significant effect on the stiffness and deformation of the nodule. The higher the thickness of the top hemisphere and the lower the thickness of the bottom hemisphere, the greater the stiffness the nodule can achieve. Similarly, the display shape of the nodule can be maintained with minimal or no deformation if the nodule top hemisphere thickness is optimally higher than bottom hemisphere thickness.

## 1. Introduction

Globally, one of the leading causes of death is cancer, and it is estimated that by 2030, the number of new cancer cases will rise up to 23.6 million per year [1]. Early diagnosis plays a very important role in prevention and consequently reduction of cancer death rates. The earlier it is diagnosed, the better the likelihood of survival of a cancer patient. Palpation is one of the fundamental steps used by physicians for routine examinations or early diagnosis of diseases such as physical inspection to detect possibly malignant tumors that can be developed underneath the skin by feeling the abnormality and assessing how stiff it is [2,3,4,5,6].

Malignant tumors are relatively stiffer than normal tissues located inside a compliant tissue as a rigid mass [5,7,8,9]. Differences in size and stiffness of a tumor may provide information about the early initiation of the disease or its severity, and a physician’s expertise is critical to distinguish the severity of the tumor [2,4,7,8,10]. Conventionally, once a patient senses an abnormality or lump underneath their skin, the patient would need to visit a physician to identify it. As a fundamental and preliminary step of diagnostics, the physician would palpate the region of interest to identify the abnormalities of the lump. This conventional method of early diagnosis procedure can be enhanced, and physical visit of a patient can be minimized if a tactile display device can recreate the lump of a patient enabling remote palpation.

As reported in the tactile display literature, currently there are several devices that attempt to emulate touch feedback utilizing haptics, tactile, or imaging [11,12]. The tactile visual images are used to produce pseudo haptics with the existing technology [13,14]. Commercially, numerous physical palpation simulators are available for clinical training purposes mainly for abdominal assessment and breast examination model [15,16,17,18]. In essence, these simulators are soft organ models with some fixed number of interchangeable organ inserts to reproduce the palpation feeling of humans. None of these commercially available palpation training simulators have the ability or feature of controlling tissue stiffness to differentiate various levels of human tissue stiffness. A number of virtual tactile display simulators for medical palpation have been introduced in research including systems with conventional haptic devices [19,20,21]. In [19], PHANToM 3.0 commercial haptic interface with virtual reality feature is used to provide position interactions by the trainee to simulate the back of a live human subject in real time. Haptic augmented reality-based breast cancer palpation training simulator is discussed in [20], where a breast model made of soft silicone is augmented with a harder virtual tumor rendered inside the model. A bovine rectal palpation simulator for training veterinary students is introduced in [21], where a trainee will be able to palpate virtual bovine reproductive tract receiving feedback from a PHANToM haptic device. A tactile display introduced by Ishizuka et al. used magnetorheological fluid to reproduce tissue stiffness distribution [22]. Their study is limited to endoscopic palpation procedures, where stiffness can be sensed by touching the tactile surface created using an MR fluid and a permanent magnet [22]. Some of the studies are performed to introduce pneumatic simulators that use controllable air pressure for physically simulating soft tissue stiffness [23,24,25]. Moreover, a prostate tumor palpation simulator is proposed on the basis of pneumatic and augmented haptics in [26]. A combination of granular jamming and pneumatics have been utilized in a multi-finger haptic palpation device to show its superiority over single-point feedback [27]. This device has been investigated to be remotely operated in a minimally invasive surgical environment using a master–slave robotic control system. Even in the surgical environment it is common to have haptics/tactile feedback for tele-operated master–slave surgical robots [27,28,29] to facilitate the surgeons but it is not yet a common practice if there is nothing in telemedicine healthcare to create a feedback between the patient and the doctor in the form of haptic/tactile feedback from palpation.

From the literature review, we observed that all of the simulators mentioned above demonstrate only a few particular organs of the human body and also lack a viable and practical palpation display for primary diagnosis of a tumor without any need of human contact (physician to patient), which has been identified as one of the major issues. Existing devices and mechanisms are not capable of aping a nodule for palpation that is able to vary the stiffness of a nodule at the same time maintaining the shape of the same nodule. Moreover, the capability of providing early diagnosis of tumor through a remote palpation facility is still a missing component in existing systems in telemedicine.

To further investigate in this way and to enhance the early diagnosis procedure, we introduced a concept and designed a simplified and compact palpation device that would facilitate a physician with the physical experience of variable stiffness level of a lump so that they can differentiate among a cyst, possibly malignant; premalignant tumor; and benign tumor through utilizing the device [30,31,32]. By further development, the device will have the facility to be connected through remote network to acquire the lump information from a patient’s body and recreate/simulate the lump in the device at the physician’s end. Thus, as an extended function of telemedicine, a physician can utilize the proposed device to be able to physically experience the lump remotely for early diagnosis of a cancerous tumor. Therefore, as a broader application, the proposed display unit can fulfill the gap in remote healthcare systems.

The device can be integrated with telemedicine system for general, rural, elderly, and other non-emergency healthcare enabling physicians to receive similar sensation of palpation from remotely located patients, creating a near in-person contact environment. The current global pandemic has limited the regular patient visits to the physicians to eliminate physical contact and reduce the spread of the COVID-19 virus unless there is an emergency or critical condition [33,34]. As a result, basic medical care is being hindered for several months. This raised an alarm that, for such an unprecedented and unexpected situation, the whole world’s medical facilities are not only underprepared to deal with emergency situations but also have limited capacity to deal with non-urgent/non-emergent patients who need regular/special medical attention. For instance, initially, regular diagnosis of lump or tumor might seem to be a non-emergency, but now it is not getting enough attention since at present the diagnosis and prevention of the spread of COVID is the priority. Due to less attention being paid to primary diagnosis facilities, a tumor can be undetected as a life-threatening cancer. In such a situation, we deem our proposed palpation display and experimental feasibility study in its uniquely designed nodule prototype can contribute as a small but fruitful step forward towards building an efficient healthcare system that allows for the ability of contactless tumor diagnosis. Along with the integration of the telemedicine concept we proposed earlier [32], the palpation device could allow for the ability to provide a solution for contactless early diagnosis for tumor detection and remote healthcare without any threat to the patient or the community. A secondary application of this proposed palpation display device could be a training apparatus for a new practitioner to have a physical palpation experience and to learn how to differentiate and diagnose diseases.

In our previous publications, we introduced our concept and overall design of the palpation display containing a nodule capable of varying levels of stiffness and at the same time maintaining its shape, structural analysis, and quick prototyping of the designed nodule for a preliminary feasibility study [30,31]. This paper explains our nodule manufacturing approach, extends the feasibility study further and explores several iterations of the nodule prototype for the palpation display, experimentally evaluates mechanical characteristics (i.e., stiffness, deformation) in testing several prototypes, and discusses future work on the basis of the experimental results. In this paper, we limited the scope of the experiments to nodule prototype with varying thicknesses to study its effect on stiffness and its deformation for the palpation display as an output device. Nevertheless, this device can be incorporated with additional supporting modules allowing user input.

## 2. Materials and Methods

Typically, a physician diagnoses and predicts whether the lump is malignant or benign by palpating its stiffness from the top surface. Two different tumors may have similar shapes but the difference in stiffnesses reveals the severity of a tumor during primary diagnosis through palpation. Therefore, our goal is to design a nodule that is capable of varying its stiffness while maintaining its shape on the display (top) surface. To incorporate this property in a nodule, we designed the palpation display integrating soft robotics and granular jamming techniques to change the stiffness of the nodule that can be perceived by palpating on the display side. Since palpation can only be performed on the skin side of the nodule, our target was to control the stiffness and maintain the shape of the upper portion of the nodule. The palpation display device consists of a nodule, whose stiffness is controlled using a pneumatic controller and granular jamming.

### 2.1. Experimental Nodule Prototypes Design

**Figure 1:**
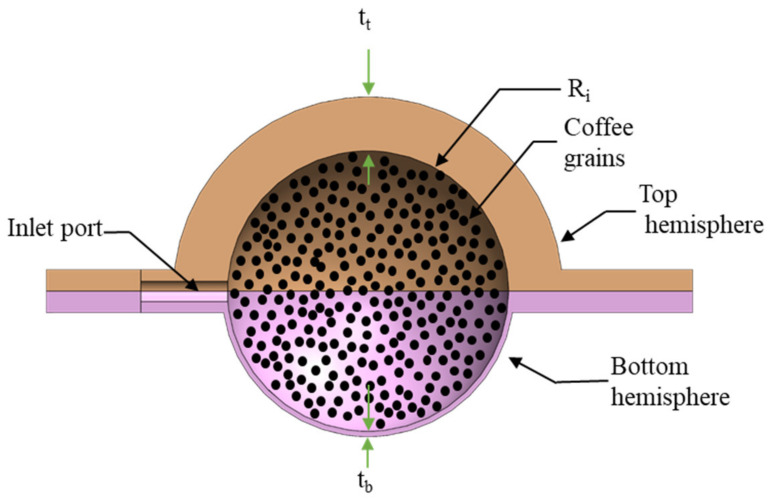
CAD model of the nodule with coffee grains showing different wall thicknesses of the top and bottom hemisphere (t_t_, t_b_, and R_i_ represent thicknesses of the top and bottom hemispheres and radius of the inner space of the nodule, respectively).

For the palpation display nodule, the granular jamming technique is an important part of our stiffness control with a modification from its traditional approach. In traditional granular jamming, grains are enclosed in a homogeneous enclosure. The limitation with the traditional approach is that grains are enclosed in a uniform body, and thus when vacuum is applied, the body decreases in volume, resulting in a shape change (deformed) of the full nodule. To overcome this issue and to maintain shape while controlling the stiffness on the display side, we designed the nodule as a combination of two hemispheres so that the top hemisphere can preserve the shape while the bottom hemisphere can respond more to the pressure change. We achieved this goal by integrating two different hemispheres with different thicknesses (Figure 1). It allowed for the thinner bottom portion of the nodule to deform under vacuum pressure.

The detailed working principle and mechanism of the nodule was introduced in our previous study [30,31]. Under vacuum pressure, all the force components inside act towards the center. However, since the bottom hemisphere is deforming more, in the fully deflated condition, all the forces act towards the top hemisphere, providing the required stiffness while maintaining the shape on the display side. To keep the manufacturing process simple and reliable, we chose soft 3D printing technology to print custom-made soft hollow hemispheres of different iterations using Agilus30™ from Stratasys, a superior rubber-like PolyJet™ material for rapid prototyping and advanced design verification with the Shore Hardness of 30 [35].

**Figure 2:**
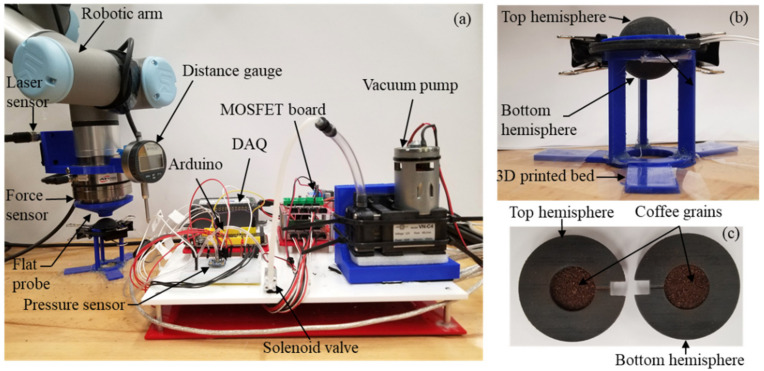
Experimental setup consisting of (**a**) robotic arm, a force sensor, flat probe, a laser sensor, distance gauge, control board (Arduino, MOSFET board, pressure sensor and solenoid valve), vacuum pump, data acquisition system (DAQ). (**b**) Soft 3D-printed granular particle-filled nodule prototype (top hemisphere and bottom hemisphere) positioned on a 3D printed bed with a circular pocket. (**c**) Internal view of the nodule filled with coffee grains.

To ease the assembly, we 3D printed two hollow flexible hemispheres with an extended radial flap around its radius (Figure 2). We inspected the 3D-printed parts for any kind of defect that may compromise the structural integrity of the nodule. Since we printed our prototypes in two parts (i.e., top and bottom hemisphere), we were able to inspect the outer and internal surfaces thoroughly. Moreover, we used a micrometer to measure the thickness and digital slide calipers to verify the dimensional parameters. After inspection, the flaps were glued together, creating a hollow spherically shaped soft nodule that was filled with granular material and connected to a vacuum pump through an inlet port. The flaps were incorporated in the design to simplify the 3D printing process of the nodule, creating a simple interface to attach the two hemispheres of similar or different thicknesses. This also eliminated the complex process of printing hollow nodules from one single print and provided more easier access to include the granular materials inside the nodule.

To keep the volumetric space constant for each nodule prototype, we maintained the inner diameter of the hemispheres constant, but to vary thickness, we changed the outer diameter of each hemisphere of the nodule, as shown in (Figure 1). There were two advantages in this approach. First, we were able to attach any two combinations of hemispheres together, irrespective of their outer thicknesses. This ensured that the amount of granular material remained constant for all the experimental prototypes and ensured the applied force on the nodule solely was absorbed by the grains and the internal surface of the nodules. This design approach assisted us in maintaining our experimental protocol on every nodule in a similar fashion.

Comparison studies on different granular particles used for granular jamming in soft robotics revealed that the variability in ground coffee grain shapes help to better jam pack them together under a vacuum condition [36]. Therefore, we used ground coffee grains as granular material for our nodule. Ground coffee contains particles of millions of different sizes. Typically these different sized particles include sizes below 100 microns to around 1700 microns [37]. These particles are relatively accommodated in a few micron ranges resulting in grind distribution. The nodule chamber was filled with coffee grains within its capacity so that the nodule felt pliable, but not to the point of overstuffing. We used ultra-soft tissue paper as filter in order to prevent the coffee grains from being drawn into the pneumatic pipeline and simultaneously not to interfere with the overall stiffness.

### 2.2. Vacuum Controller

Two spherical hemispheres were attached together, forming the nodule, and a small circular inlet was added for inserting the pneumatic pipe. The nodule was connected with a vacuum pump via a two-way solenoid valve. A pressure sensor was placed in between the solenoid valve and the inlet of the nodule with a pneumatic pipe (Figure 2). The pressure sensor (MicroPressure MPR Series, Honeywell, Inc., Charlotte, NC, USA) was chosen as it could measure absolute pressure (0–25 PSI). The pressure sensor measured the pressure inside the nodule and once the nodule reached certain vacuum pressure, the two-way solenoid valve closed and diverted the air flow to open space to avoid the pump from stalling. A microcontroller (Arduino mega 2560, Keyestudio, Shenzhen, China) read the pressure sensor data and controlled the solenoid valve and the vacuum pump accordingly with the help of a MOSFET control board (DAOKI, Shenzhen, China). At atmospheric pressure, the nodule felt soft and pliable. When vacuum pressure was applied, the coffee grains inside the nodule chamber locked themselves together, creating a stiff nodule.

### 2.3. Experimental setup

Figure 2 shows the setup for the experiment. The setup consisted of a UR3e robotic arm (Universal Robots, Inc., Odense, Denmark), an ATI Axia80 force sensor (ATI Industrial Automation, Inc., Apex, NC, USA), a distance laser sensor (OADM 20I2472/S14C, Baumer Ltd., Frauenfeld, Switzerland), a control board (consisting of the Arduino board, MOSFET board, pressure sensor, and solenoid valve), a data acquisition system (DAQ), and a laptop. ATI Axia80 force sensor was incorporated at the end effector of the UR3e robotic arm to measure the stiffness of the nodule. A 3D-printed flat probe of 10 mm diameter was mounted in front of the force sensor to maintain uniform contact surface area throughout the experiment. The robotic arm was used to indent the nodule with high precision along the vertical axis. The nodule was positioned on a 3D-printed bed with a circular pocket, where it rested on the extended flaps of the nodule. This specific support around the extended flap ensured that the force exerted by the robotic arm was only absorbed by the nodule and not by the surroundings. It also provided an elevation for the nodule so that the bottom part of the nodule was not in contact with other surface where the exerted force by the robotic arm can be absorbed. The distance laser was incorporated with the arm along with the ATI force sensor to measure the distance when the robotic arm started the indentation process. Moreover, a digital distance gauge was attached on the end effector of the robotic arm. This gauge was used to measure the vertical deformation of the display side of the nodule.

### 2.4. Experimental Evaluation and Protocols

It was hypothesized that the nodule shape on the display side would be able to be maintained even after vacuuming and to achieve its highest stiffness state. It was also hypothesized that while the vacuum was performed to vary the stiffness, the display side (top hemisphere) would have minimal to no deformation.

- Since one of the important features of our design included different thicknesses of two hemispheres of the nodule, we set a number of combinations by altering the wall thickness of the top and bottom hemispheres to study and evaluate the behavior of the nodule under uniform vacuum pressure. We 3D printed the top hemispheres of the nodule with thicknesses of 5, 4, 3, and 2 mm and bottom hemispheres with thicknesses of 5, 4, 3, 2, 1, and 0.5 mm.
- The thickness combinations we have chosen to assemble the nodules for this experiment are as follows (top hemisphere-bottom hemisphere):
  ▪ 5 mm-5 mm, 5 mm-4 mm, 5 mm-3 mm, 5 mm-2 mm, 5 mm-1 mm, 5 mm-0.5 mm;
  ▪ 4 mm-4 mm, 4 mm-3 mm, 4 mm-2 mm, 4 mm-1 mm, 4 mm-0.5 mm;
  ▪ 3 mm-3 mm, 3 mm-2 mm, 3 mm-1 mm, 3 mm-0.5 mm;
  ▪ 2 mm-2 mm, 2 mm-1 mm, 2 mm-0.5 mm.
- Each combination of the top and bottom hemisphere varied only by thickness but the internal hollow space (i.e., volume) remained the same. As mentioned earlier, the internal hollow space was filled with equal amount of coffee grains and sealed together to assemble each nodule.
- First, we measured the stiffness of each nodules under no-vacuum (1 atmospheric pressure) condition.
- Later, we measured the stiffness under vacuum condition (2 ± 0.3 PSI).

### 2.5. Experimental Data Collection Method

The reaction force exerted by the nodule under normal (not vacuumed) condition and vacuumed condition was recorded. When the nodule was under normal atmospheric pressure, the indentation and force measurement process was repeated five times to record five trials of measurement data. For each trial, the nodule was indented until 5 mm in depth in total five steps (1 mm in each step). Under vacuum condition, when coffee grains were jammed, stiffening the nodule, the first trial of measurement was taken. After each trial, we repeated the whole process by stopping the vacuum pump to ensure that the nodule returned to its initial condition. Since we had a pressure sensor connected with the nodule, we were able to monitor the pressure inside the nodule. Once it reached the atmospheric pressure, we also inspected the nodule visually to ensure that it reached its initial condition. Once the granular particles were packed under vacuum, they might still have been jammed together. To ensure that the nodule was in its initial condition and the grains were not jammed, we applied gentle massage around the nodule for loosening the grains before each trial. This process was repeated for all five trials while collecting force measurement data under vacuumed condition.

We were also interested in observing the deformation on the display side of the nodule. To measure the deformation of the display side, we used a distance gauge (DIGR-0105, Clockwise Tools, Inc., Valencia, CA, USA). The gauge was placed on the tip of the nodule on the display side under no-vacuum and vacuum conditions using the help of the robotic arm (Figure 2). To observe the change in shape of the granular particle-filled nodule under uniform vacuum pressure and varying thickness of the top and bottom hemispheres, we mounted the digital distance gauge on the end effector of the robotic arm. The thickness combinations were kept the same as the first experiment. The digital distance gauge was placed on the top-middle portion of each nodule using the arm. This spring-loaded distance gauge was used to measure the deformation of the nodule before and after vacuum condition.

We also kept a visual record of the deformation of each nodule to understand the deformation pattern of different nodules. We measured the height change of the nodule before and after applying the vacuum pressure. It was expected that under applied vacuum pressure, coffee grains were jammed, and if the bottom hemisphere was thinner as compared to the top one, it would deform first, pushing the jammed coffee grains towards the top. Hence, the shape change would be lesser as compared to the same thicknesses of top and bottom hemispheres, keeping the display side undeformed or minimally deformed. In this experiment, we observed and compared the vertical deformation (height measurements) taken for the nodule with several combinations of thicknesses chosen as top and bottom hemispheres.

## 3. Results

To evaluate the design and feasibility of the nodule, we needed to see how the differences in wall thickness were able to impact in the stiffness of the nodule both under normal (non-vacuum) and vacuum conditions. In our experiment, we varied the thicknesses of the top and bottom hemispheres to test the behavior of the nodule under normal (non-vacuum) and vacuum conditions.

### 3.1. Nodule Stiffness Test Varying Thickness of Top and Bottom Hemispheres under No-Vacuum (No-Vac) Condition

**Figure 3:**
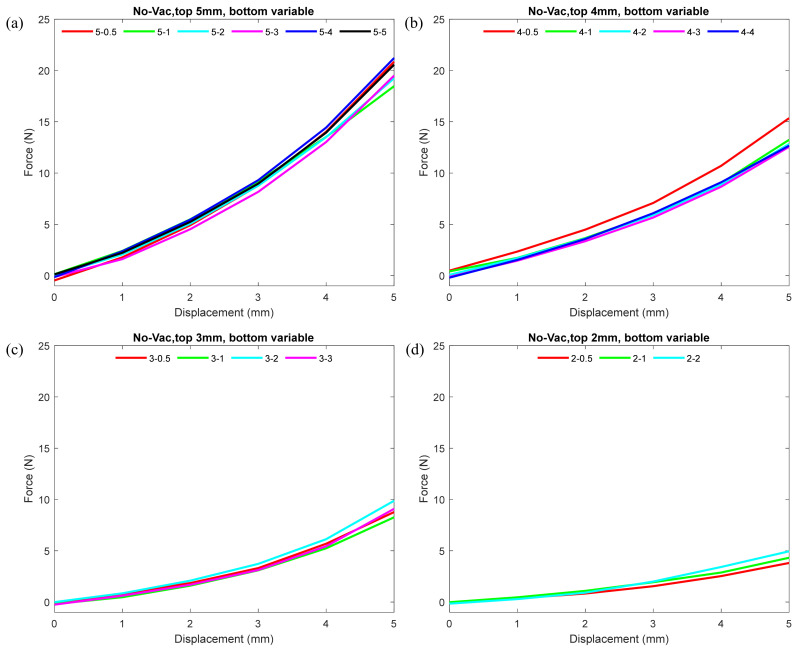
Force–displacement curves showing the effect of different top and bottom hemisphere wall thicknesses of the nodule on its stiffness under normal (not vacuumed) conditions. (**a**) Plots including top hemisphere wall thickness 5 mm and bottom hemisphere wall thicknesses varying from 5 to 0.5 mm. (**b**–**d**) Plots including top hemisphere wall thicknesses 4, 3, and 2 mm, respectively, and bottom hemisphere wall thicknesses varying from 5 to 0.5 mm. For detailed individual trial data including standard deviation, please refer to the scatter plots in Figure A1 in the Appendix A.

To observe the effect of different thicknesses of the nodule on its stiffness under normal conditions, we plotted force–displacement curves, as shown in Figure 3. The plot demonstrates how the variation in the wall thicknesses of the top and bottom hemisphere contributed to the stiffness of the nodule under non-vacuum condition. In each plot in the figure, the top hemisphere thickness is kept constant, and the thickness of the bottom hemisphere varies gradually at 0.5 mm intervals. Note that in every comparison, one combination is included where the bottom and top hemisphere wall thicknesses were equal, and in none of the combinations did the bottom hemisphere wall thickness exceed the top hemisphere. From each plot in Figure 3, one can see that, under non-vacuum condition for each top hemisphere, the force–displacement values were almost identical, regardless of the variation in the wall thickness of the bottom hemisphere. The configurations with the thickest (5 mm) top hemisphere displayed maximum stiffness, and the thinnest (2 mm) displayed minimum stiffness.

### 3.2. Stiffness Test Varying Top Hemisphere and Keeping Bottom Hemisphere Constant under Vacuum (Vac) Condition

**Figure 4:**
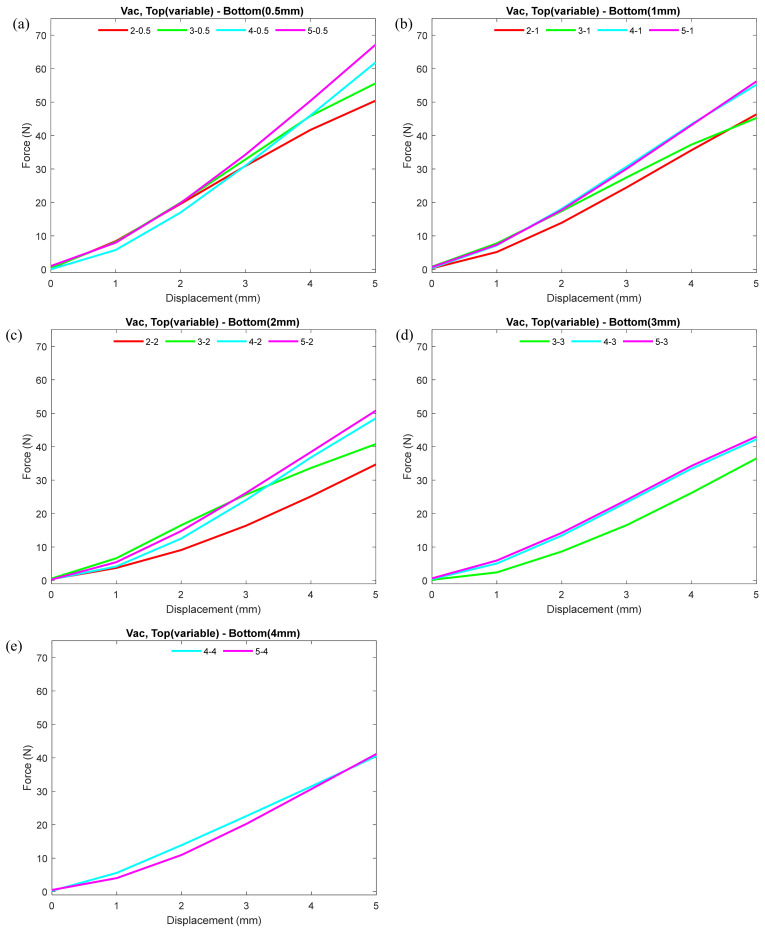
Force–displacement plots showing the effect of change in top hemisphere wall thickness while keeping the bottom hemisphere wall thickness of the nodule constant under vacuumed condition. For each plot, the different colors correspond to different top hemisphere thicknesses (i.e., 5, 4, 3, and 2 mm), while the bottom hemisphere wall thickness is kept constant. (**a**) Plots where bottom hemisphere wall thickness was kept at 0.5 mm. (**b**–**e**) Plots where bottom hemisphere wall thicknesses were kept at 1, 2, 3, and 4 mm, respectively. For detailed individual trial data including standard deviation, please refer to the scatter plots in Figure A2 in the Appendix A.

**Figure 5:**
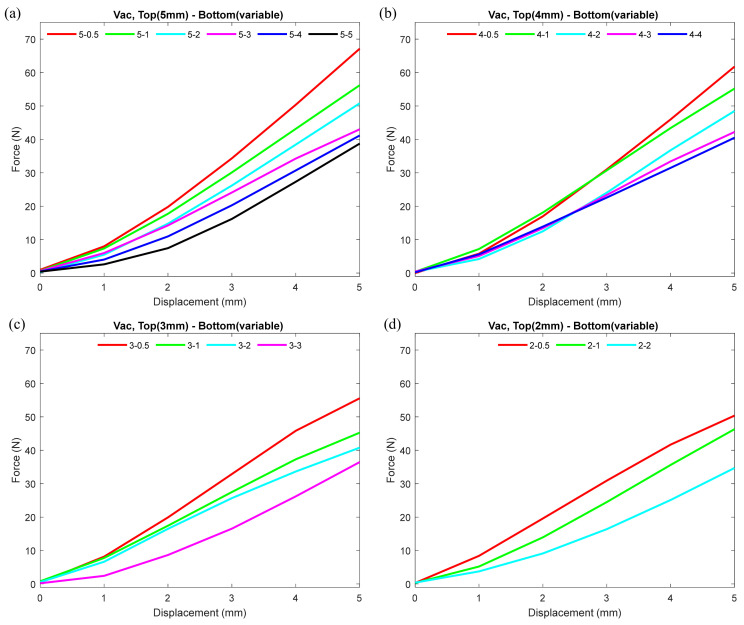
Force–displacement curves showing the effect of change in bottom hemisphere wall thickness while keeping the nodule top hemisphere wall thickness constant under vacuumed condition. For each plot, the different colors represent different bottom hemisphere thicknesses (i.e., 5, 4, 3, 2, 1, 0.5 mm), while the top hemisphere thickness is kept constant. (**a**) Plots where top hemisphere wall thickness was kept at 5 mm. (**b**–**d**) Plots where top hemisphere wall thicknesses were kept at 4, 3, and 2 mm, respectively. For detailed individual trial data including standard deviation, please refer to the scatter plots in Figure A2 in the Appendix A.

To observe the effect of various thicknesses of the bottom and top hemispheres on the nodule stiffness at a constant vacuum pressure (2 ± 0.3 PSI), we presented the experimental data in two different manners, as shown in Figure 4; Figure 5. Figure 4 shows the comparison of force–displacement plots where the wall thickness of the bottom hemisphere of the nodule remained constant and the top hemisphere thickness varied. For each plot in Figure 4, the different colors represent the different top hemisphere thicknesses (i.e., 5, 4, 3, and 2 mm). The plot helps to identify the effect of change in the top hemisphere thickness for the nodule while the bottom hemisphere remains constant. Note that for the comparison plot, the bottom hemisphere thickness never exceeded the top hemisphere thickness, since the bottom hemisphere exceeding the top hemisphere thickness will cause deformation on the display side of the nodule. A pattern can be observed from each plot in that the higher the thickness of the top hemisphere, the greater the stiffness. For all the cases shown in Figure 4, the nodule with the greatest thickness (5 mm) for the top hemisphere exhibited the largest force, and the top hemisphere with the least thickness (2 mm) demonstrated the minimum force. Additionally, the case with the thinnest (0.5 mm) bottom hemisphere combination exhibited the largest force among all the combinations.

### 3.3. Stiffness Test Keeping Thicknesses of Top Hemisphere Constant and Varying Bottom Hemisphere under Vacuum (Vac) Condition

Figure 5 presents the comparison of force–displacement curves while the thickness of the top hemisphere remained constant and the thickness of the bottom hemisphere varied. For each plot in Figure 5, the different colors represent different bottom hemisphere thicknesses (i.e., 5, 4, 3, 2, 1, 0.5 mm), while the top hemisphere thickness is kept constant. The plot helps to identify the effect of change in bottom hemisphere thickness for the nodule while the top hemisphere remained constant. Note that in the comparison, the top hemisphere thickness never exceeded the bottom hemisphere thickness. It can be observed that the thinner the bottom hemisphere, the higher the stiffness of the nodule. The thinnest (0.5 mm) bottom hemisphere contributed to the highest stiffness value.

### 3.4. Change in Stiffness between No-Vac and Vac Conditions

**Figure 6:**
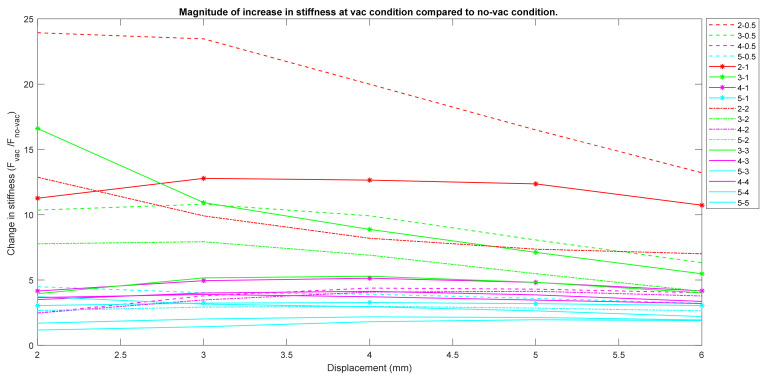
Magnitude of increase in nodule stiffness at vacuum condition compared to non-vacuum condition.

Figure 6 demonstrates a comparison plot showing the magnitude of increase in stiffness at vacuum condition compared to non-vacuum condition for each nodule combination. Each color indicates a certain thickness of top hemisphere, and the different legends in the same color indicate a different bottom thickness. Nodules associated with the higher top hemisphere thickness (i.e., 5 mm and 4 mm) had the lowest magnitude of increase in stiffness. Nodules with 4 and 5 mm top hemisphere thickness had 1- to 4-fold increase in stiffness, which was lower compared to the other nodule combinations with 2 and 3 mm top hemisphere thicknesses. Nodules with top thickness of 2 and 3 mm with thinner bottom hemisphere exhibited five- or higher fold increase in stiffness. Nodules with 2 mm top and 0.5 mm bottom hemisphere exhibited the largest stiffness increase of 15- to 25-fold. All the combinations of nodule with 2 and 3 mm top hemisphere with thinner bottom hemisphere had a downward stiffness change, while the nodules with thicker top hemisphere (5 and 4 mm) had mostly a horizontal curve.

### 3.5. Deformation of the Nodule

Table 1 presents the vertical deformation of the nodule from the display side for three measurements (trials 1, 2, and 3) shown in each row using distance gauge as mentioned earlier. The mean value in the bottom row is representative of all the possible combinations except the nodules with similar top and bottom hemisphere wall thickness. For same-thickness top and bottom hemisphere combinations, we observed large and random variations in vertical deformation. Nodules with bottom hemisphere of 0.5 mm and top hemisphere of 2 and 3 mm demonstrated similar deformation of about 2 mm, whereas nodules with bottom hemisphere of 0.5 mm and top hemisphere of 4 and 5 mm demonstrated similar deformation of about 1 mm. Nodules with thicker top hemisphere than the bottom hemisphere displayed higher vertical deformation as the thickness of the bottom hemisphere increased, except we saw a minor exception in the case of nodules with 3_1. Even in this case, the mean was very close to its adjacent left column and had a difference of only fraction of a millimeter.

#### Visual Record of Nodule Deformation Results

**Figure 7:**
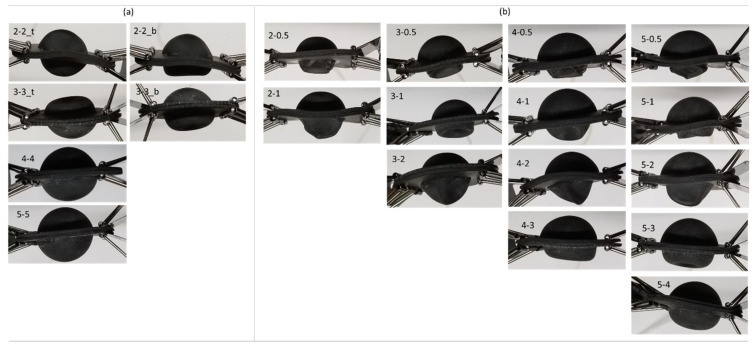
Visual record of nodule deformation under vacuum pressure for (**a**) nodules with same wall thickness on top and bottom hemispheres, and (**b**) nodules with top hemisphere that contain higher wall thickness compared to their bottom hemisphere.

We also kept a visual record of the deformation of each nodule to understand the deformation pattern of different nodules. Figure 7 shows the deformation images under vacuum pressure for each combination of top and bottom hemisphere thicknesses. Figure 7a shows cases where the nodule had similar wall thickness on both top and bottom hemispheres: left- and right-side images of Figure 7a show the same nodule in two different random trials. Except for top–bottom hemisphere thickness of 4 mm-4 mm and 5 mm-5 mm, random trial results were visually similar from top or bottom hemisphere sides, and therefore single images are shown. The left side images show deformation observed on the display side and the right-side images show deformation observed on the bottom of the nodule. Figure 7b shows nodules with a thicker top hemisphere than bottom hemisphere. For the nodules with thinner bottom hemisphere, the deformation was always observed on the bottom, keeping the display side (top hemisphere) with minimal or no deformation. Visually, in case of thicker top hemisphere as compared to bottom, we observed a very negligible amount of shape change on the display side of the nodule. However, only when both top and bottom hemisphere thicknesses were kept same did we observe a visual deformation on the top hemisphere.

## 4. Discussion

In this article, we presented a feasibility study and experimental evaluation of a uniquely designed nodule developed for our palpation display apparatus, a novel apparatus that we introduced to facilitate non-contact primary diagnosis of tumor through palpation. Two different hollow hemispheres of different thicknesses were attached together, and with the help of granular jamming, we demonstrated experimentally that our mechanism is capable of changing its stiffness while maintaining its shape on its display side. This paper focused on testing the stiffness capability of our designed nodule and evaluating its behavior on the basis of its stiffness and deformation. In this paper, we explored several combinations of thicknesses for top and bottom hemispheres, experimentally evaluated each combination to test the mechanical properties (i.e., stiffness, deformation) of the nodule, compared the force–displacement data, and identified the impact of the top–bottom hemisphere thickness difference on the behavior of the nodule.

Figure 3 indicates that under non-vacuum condition for a fixed top wall thickness irrespective of its bottom hemisphere thickness, the nodule stiffness remained nearly identical. In normal (non-vacuum) condition, granular particles inside the nodule were loose, and therefore were able to flow freely. As a result, the nodule was soft and pliable. At this point, the grains provided less of a resistance to the external force while indenting. The loosely packed grains accommodated the space for the indentation. Therefore, the wall thickness of the top hemisphere primarily contributed to the stiffness of the nodule under normal conditions. Results from Figure 4; Figure 5 both indicated that the top hemisphere with a higher thickness exhibited higher stiffness.

The vacuum pressure acted on the nodule’s internal surface and compressed the grains. Therefore, the grains were jammed, and these jammed coffee grains contributed additionally to the top hemisphere stiffness at vacuum condition. As a result, top hemisphere thickness with 2 and 3 mm showed lesser stiffness compared to 4 and 5 mm. The stiffness between top hemisphere with 4 and 5 mm thicknesses were close to each other. This was due to greater thickness leading nodules to respond less to the vacuum pressure. Moreover, from the results, we observed that lowering the wall thickness of the bottom hemisphere contributed to achieving the higher stiffness of the nodule under vacuumed condition. Nodules with thinner bottom hemisphere contributed to higher stiffness because the thinner wall provided less structural restriction and deformed more under vacuum pressure. This lower restriction resulted in higher pressure on the coffee grains towards the inner surface of the top hemisphere. Moreover, the thinner bottom hemisphere deformed first towards the top, and in fully deflated condition, all the forces acted towards the top hemisphere, contributing to higher stiffness while maintaining shape on the display side. This push, similar to a syringe’s plunger effect, caused higher force on the interior surface of the top hemisphere wall, which resulted in increased stiffness.

The plot from Figure 6 showing the magnitude of increase in stiffness at vacuum condition from its non-vacuum condition also validates that the nodules with thinner bottom hemisphere always exhibited several folds of stiffness increase. For the same reason, the nodules with thinner bottom hemisphere combinations exhibited superior increase in stiffness in comparison with the thicker ones. The nearly horizontal curve in Figure 6 regarding 4 and 5 mm top hemisphere thickness indicates that the increase in stiffness was almost constant for every indentation depth. This was because 5 and 4 mm top hemispheres were significantly thick and were stiffer than other hemispheres on their own. Thus, the change in bottom hemisphere thickness did not impact the stiffness significantly. On the other hand, the thinner top hemispheres (i.e., 2 and 3 mm) were structurally less stiff, and the structure itself contributed less to its own stiffness.

For the nodules with 2 and 3 mm top thickness, the curve went downwards after certain indentation depths. This was because, when the nodule was indented, after certain indentation steps, the bottom hemisphere was unable hold its position firmly and deformed downwards due to the external force applied on the top hemisphere. At this point, the nodule loses its stiffness. For comparison study and in order to provide further insight, we in our experiments considered indentation depts until 5 mm. However, since the nodule would be used for a palpation display, we assumed that palpation depth of 5 mm would most likely not be required. Rather, a palpation depth of 2–3 mm would be sufficient for the healthcare personnel to perform palpation.

We observed uniform deformation (if any) on the display side when the nodule had thinner bottom hemisphere than the top, as shown in Figure 7, which supports our hypothesis. The nodule shape deformation was random when both top and bottom hemispheres had the same wall thickness. In theory, when the nodule has similar wall thickness throughout, it should deform in a uniform manner around all of its surface. However, in practical situations, the nodule is filled with coffee grains. Therefore, when it is being compressed by the vacuum pressure, the grains do not compress in a similar manner. At this point, a random surface point/small area inside the nodule faces higher force than its surrounding surface. Therefore, slightest deformation in the nodule will create an unpredicted overall deformation around the region.

Moreover, when the nodule is under vacuum pressure, the slightest preexisting deformation or structural weakness on the 3D printed nodule will create a pressure concentration on a certain point (i.e., surface area), which will result in deformation in that region. On the other hand, the nodules with higher thickness on the top hemispheres than the bottom hemispheres show deformation on the bottom hemisphere, preserving the shape of the display side (top hemisphere). Due to structural flexibility of the thinner wall on the bottom hemisphere, bottom hemisphere reacts more to pressure changes and deforms upward.

We observed minimum deformations for all nodules with thicker top hemisphere compared to bottom, as shown in Table 1. Since 5 and 4 mm hemisphere are significantly thick, their vertical deformations were also less under vacuum condition. The upward force from the bottom hemisphere in vacuum condition was less affected on the inner top surface of the nodule, but at the same time, its own stiffness aided the nodule to maintain its shape. For the opposite reason, in nodules with 2 and 3 mm top hemispheres, we saw a slightly higher vertical deformation. This was because the thinner walls of these nodules responded more to vacuum pressure. However, for nodules with 0.5 mm bottom hemisphere, these vertical deformations were not very significant and had a variation of about 1 mm.

We believe that the small variation in the pattern of Table 1 regarding vertical deformation for nodule 3_1 was a minor mechanical error of the distance gauge we used. Since the gauge has a spring-loaded tip for distance measurement, this spring force may have caused the minor indentation error. In our future work, we plan to utilize surface mapping to study the overall shape deformation in more detail. Nevertheless, the mean of nodule 3_1 was very close to its adjacent left column of Table 1 and had a difference of only fraction of a millimeter. Moreover, if we consider Table 1 and Figure 7 (deformation figures), we can see that the random indent on the top hemisphere was the probable cause for the random vertical deformation for the nodule with similar thickness with top and bottom hemispheres. Due to this randomness in similar thickness combination, in Table 1, we see a comparatively larger standard deviation in the case of nodule 2_2.

As hypothesized, keeping the bottom hemisphere thinner compared to the top hemisphere assisted us in controlling the stiffness of the nodule with minimal shape deformation on the display side and at the same time assisted in avoiding the deformation randomness on the display side. On the basis of the overall experimental results and considering Figure 6; Figure 7, we contemplated top-bottom hemisphere combinations of 2 mm_0.5 mm and 3 mm_0.5 mm as optimized candidates to be used as nodules for the palpation display for stiffness control. Figure 6 (stiffness change) suggests that these two had the highest range of stiffness amplification, whereas all the nodules with 4 and 5 mm top hemisphere had a very small (bellow 5) fold of stiffness increase. Higher number in stiffness change provides a superior mechanical advantage. Moreover, these two optimally chosen nodule options had significantly low deformations on the top hemisphere. This resulted in a minimal deformation on the display side of the nodule that is beyond human perception.

To fabricate the top and bottom hemisphere of the nodule, we used only one material, i.e., Agilus30™ from Stratasys, a rubber-like PolyJet™ material. In future, we plan to explore other soft 3D printing materials that may suit our design requirements and to perform a comparison test to identify better suited material for the nodule behavior enhancement. In our current design, our scope is limited to controlling the stiffness with minimal or no deformation on the display side. From the deformation test result of different prototypes, we observed that the deformation challenge of the display side can be overcome by keeping the bottom hemisphere much thinner than the display hemisphere. Moreover, our current study was limited to only one vacuum pressure level. In the future, we intend to study the effect of different pressure levels on different nodules fabricated with different materials and thicknesses.

Even though we observed some minor deformation, as Table 1 suggests, in our future work, we plan to study how much of this deformation is perceivable by human subjects. On the basis of the study on human subjects, we plan to compare, optimize, and modify our nodule mechanism. Additionally, we aim to improve the design of our system to minimize the deformation on the display side further. One probable solution to this issue can be including a small automatic pushing mechanism to the bottom hemisphere to ensure the bottom hemisphere deforms first when the vacuum process starts without providing any additional structural support for the nodule. This will prevent any chance of irregular deformation on the display side and ensure total deformation on the bottom hemisphere, keeping the top hemisphere undeformed more effectively. The experiments provided insight into the force–displacement plots of the nodule. By comparing several thickness combinations of the prototype, we observed a pattern in the force–displacement plots. This helped us to characterize and understand the behavior of the nodule for better controllability. We leave further improvement of the nodule shape, size, and pressure level controllability and validation of a complete characterization model of the nodule for future work.

## 5. Patents

Title: “Tactile display apparatus for palpation simulation and telemedicine and methods of use”. US20200294424 patent pending.

## Figures and Tables

**Table 1:** Recordings of vertical deformation of nodules from the display side using digital distance gauge.

Nodule Wall Thickness (Top_Bottom)	2_0.5	2_1	2_2	3_0.5	3_1	3_2	3_3	4_0.5	4_1	4_2	4_3	4_4	5_0.5	5_1	5_2	5_3	5_4	5_5
Trial 1	2.15	4.04	3.77	1.34	1.15	3.86	7.00	1.32	1.75	2.14	5.29	7.30	1.25	1.22	1.54	2.36	2.71	2.92
Trial 2	2.24	3.34	7.13	1.66	1.38	4.20	7.29	1.24	1.70	2.48	5.49	7.24	1.19	1.24	1.46	2.21	2.84	2.62
Trial 3	2.42	3.26	7.23	1.77	1.37	5.02	7.28	1.28	1.79	2.44	5.44	7.09	1.16	1.22	1.54	2.38	2.80	2.82
Mean	2.27	3.54	6.04	1.59	1.23	4.36	7.19	1.28	1.74	2.35	5.41	7.21	1.20	1.22	1.51	2.31	2.78	2.78
Standard Deviation	0.14	0.43	1.97	0.22	0.13	0.60	0.17	0.04	0.05	0.19	0.10	0.11	0.05	0.01	0.05	0.09	0.07	0.15

**Figure A1:**
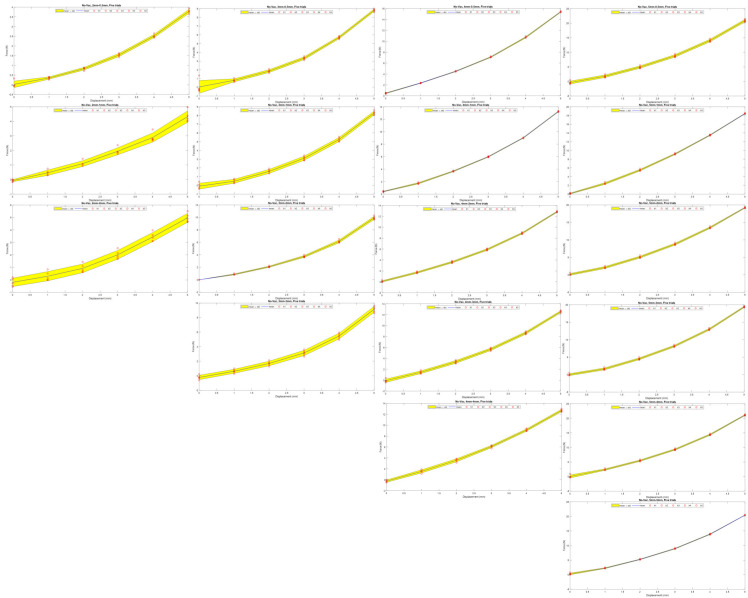
Force–displacement scatter plot showing all five trials including mean and standard deviation for each combinations of top and bottom hemisphere wall thickness of the nodule on its stiffness under normal (not vacuumed) condition. The yellow shaded area indicates ±1 standard deviation from the blue mean curve. Each red circle corresponds to a trial data.

**Figure A2:**
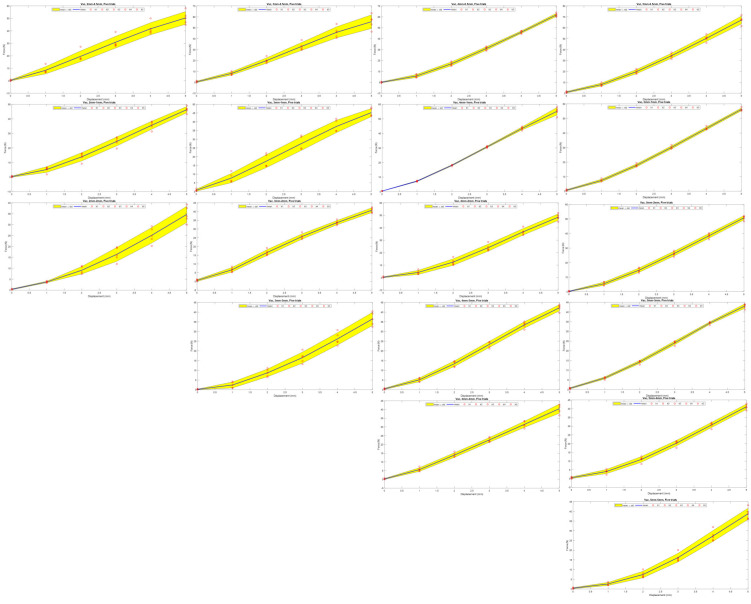
Force–displacement scatter plot showing all five trials including mean and standard deviation for each combination of top and bottom hemisphere wall thickness of the nodule on its stiffness under vacuumed condition. The yellow shaded area indicates ±1 standard deviation from the blue mean curve. Each red circle corresponds to a trial data.
